# The impact of public health interventions on the future prevalence of ESBL-producing *Klebsiella pneumoniae*: a population based mathematical modelling study

**DOI:** 10.1186/s12879-022-07441-z

**Published:** 2022-05-23

**Authors:** Luisa Salazar-Vizcaya, Andrew Atkinson, Andreas Kronenberg, Catherine Plüss-Suard, Roger D. Kouyos, Viacheslav Kachalov, Nicolas Troillet, Jonas Marschall, Rami Sommerstein

**Affiliations:** 1 Department of Infectious Diseases, Bern University Hospital, Inselspital, University of Bern, University of Bern, Freiburgstrasse, 3010 Bern, Switzerland; 2 Institute for Infectious Diseases, University of Bern, Bern, Switzerland; 3 Institute of Medical Virology, University of Zurich, Zurich, Switzerland; 4 Division of Infectious Diseases and Hospital Epidemiology, University Hospital Zurich, University of Zurich, Zurich, Switzerland; 5 Service of Infectious Diseases, Central Institute, Valais Hospitals, Sion, Switzerland

**Keywords:** ESBL-producing *Klebsiella pneumoniae*, Resistance, Mathematical model, Public health intervention

## Abstract

**Background:**

Future prevalence of colonization with extended-spectrum betalactamase (ESBL-) producing *K. pneumoniae* in humans and the potential of public health interventions against the spread of these resistant bacteria remain uncertain.

**Methods:**

Based on antimicrobial consumption and susceptibility data recorded during > 13 years in a Swiss region, we developed a mathematical model to assess the comparative effect of different interventions on the prevalence of colonization.

**Results:**

Simulated prevalence stabilized in the near future when rates of antimicrobial consumption and in-hospital transmission were assumed to remain stable (2025 prevalence: 6.8% (95CI%:5.4–8.8%) in hospitals, 3.5% (2.5–5.0%) in the community *versus* 6.1% (5.0–7.5%) and 3.2% (2.3–4.2%) in 2019, respectively). When overall antimicrobial consumption was set to decrease by 50%, 2025 prevalence declined by 75% in hospitals and by 64% in the community. A 50% decline in in-hospital transmission rate led to a reduction in 2025 prevalence of 31% in hospitals and no reduction in the community. The best model fit estimated that 49% (6–100%) of observed colonizations could be attributable to sources other than human-to-human transmission within the geographical setting.

**Conclusions:**

Projections suggests that overall antimicrobial consumption will be, by far, the most powerful driver of prevalence and that a large fraction of colonizations could be attributed to non-local transmissions.

**Supplementary Information:**

The online version contains supplementary material available at 10.1186/s12879-022-07441-z.

## Background

Over 650,000 infections with antimicrobial-resistant bacteria are estimated to occur every year in the European Union [1]. The rapid spread of extended-spectrum betalactamase (ESBL-) producing Enterobacteriaceae is of global concern [2–5]. Local and external interacting sources of colonization mediate this spread. Local sources of colonization include human-to-human transmission within communities that share geographic proximity. External sources of colonization include traveling to regions with high prevalence of colonization (high-prevalence regions), as well as consumption of contaminated food [6–8]. Transmission of ESBL-producing *K. pneumonia*, the third most prevalent resistance pathogen causing infections [1], is known to be enhanced by hospitalizations and antimicrobial consumption [7, 9, 10].

The prevalence of colonization with ESBL-producing *K. pneumoniae* in Switzerland is steadily increasing throughout the country, as evidenced by data from the Swiss Centre for Antibiotic Resistance; (ANRESIS). This prevalence is lower in Switzerland than it is in its neighbouring countries, and similar to that in the Netherlands, Norway and Denmark [11, 12].

To what extent antimicrobial consumption and local and external sources of colonization have contributed to the spread of ESBL-producing *K. pneumoniae,* and the magnitude of their effect on the future prevalence of colonization with this pathogen, is not yet understood.

This study aimed to compare the effect of different types of public health interventions on the future course of prevalence of colonization with ESBL-producing *K. pneumoniae*. We did this by means of a mathematical model calibrated to over 13 years of data on susceptibility and antimicrobial consumption from a Swiss region with stable population’s size and characteristics, and subject to incoming sources of colonization.

## Methods

### The ANRESIS database and the modelled population

Antibiotic resistance and antimicrobial consumption data were obtained from the Swiss Centre for Antibiotic Resistance database (ANRESIS), which has been previously described in detail [13]. ANRESIS prospectively collects routine and patient-specific antibiotic resistance data representing around 80% of annual hospitalization days across Switzerland. Most laboratories gather data from multiple hospitals, ranging from primary- to tertiary-care institutions and from the community (general practitioners).

Our mathematical model used data from the Swiss Canton of Valais. This canton is relatively isolated geographically and has a population of approximately 350,000 inhabitants, which remained stable throughout the observed years [14]. All 25 healthcare institutions in the Valais participate in ANRESIS, with five public hospitals contributing most of the data. Data have been collected since 2004 using standardized methodology [15].

*Antimicrobial resistance* We used the outcomes of susceptibility tests to ceftriaxone as surrogate for presence/absence of ESBL-production. We considered all clinical samples of invasive (from a usually sterile site) and non-invasive *K. pneumoniae*, and assumed invasive samples to represent clinically significant infections, and non-invasive samples to represent non-clinically significant infections and/or colonization. Clinically significant infections were assumed to lead to antimicrobial therapy. We included susceptibility data collected between 2004 and 2017. In order to avoid double counting, estimations of prevalence excluded repeated tests with the same ceftriaxone resistance results for the same patient within the same year.

*Antimicrobial consumption* In 2017 56% (69/123) of pharmacies in the region reported their data to ANRESIS. However, we had no access to the data necessary to estimate the share of antimicrobial consumption in the community captured by these pharmacies. We therefore relied on a simplified approach with simulations assuming that recorded outpatient data included 50% of all of prescribed antibiotics. Inpatient data included data from all public hospitals. Inpatient antimicrobial consumption was imputed for the smaller, private institutions by assuming that consumption per yearly bed-days equalled that in public hospitals. Data collection spanned between 2006 and 2015 for inpatients and between 2013 and 2016 for outpatients. Consumption data was expressed in defined daily doses (DDD) for the 5th level of hierarchy according to the WHOCC-ATC classification [16].

The model considers three types of antimicrobial therapy termed *regular, restricted* and *neutral*. *Regular* and *restricted* types represent no activity and activity against ESBL-producing pathogens, respectively. The *neutral* type represents antibiotics that neither select for nor have a clinical relevant effect on ESBL-producing pathogens, or the effect on ESBL producing pathogens is disputed, such as penicillin/betalactame inhibitor combinations [14, 16, 17]*.* Additional file 1: Table S1 summarizes these categories.

### Mathematical model structure

**Fig. 1:**
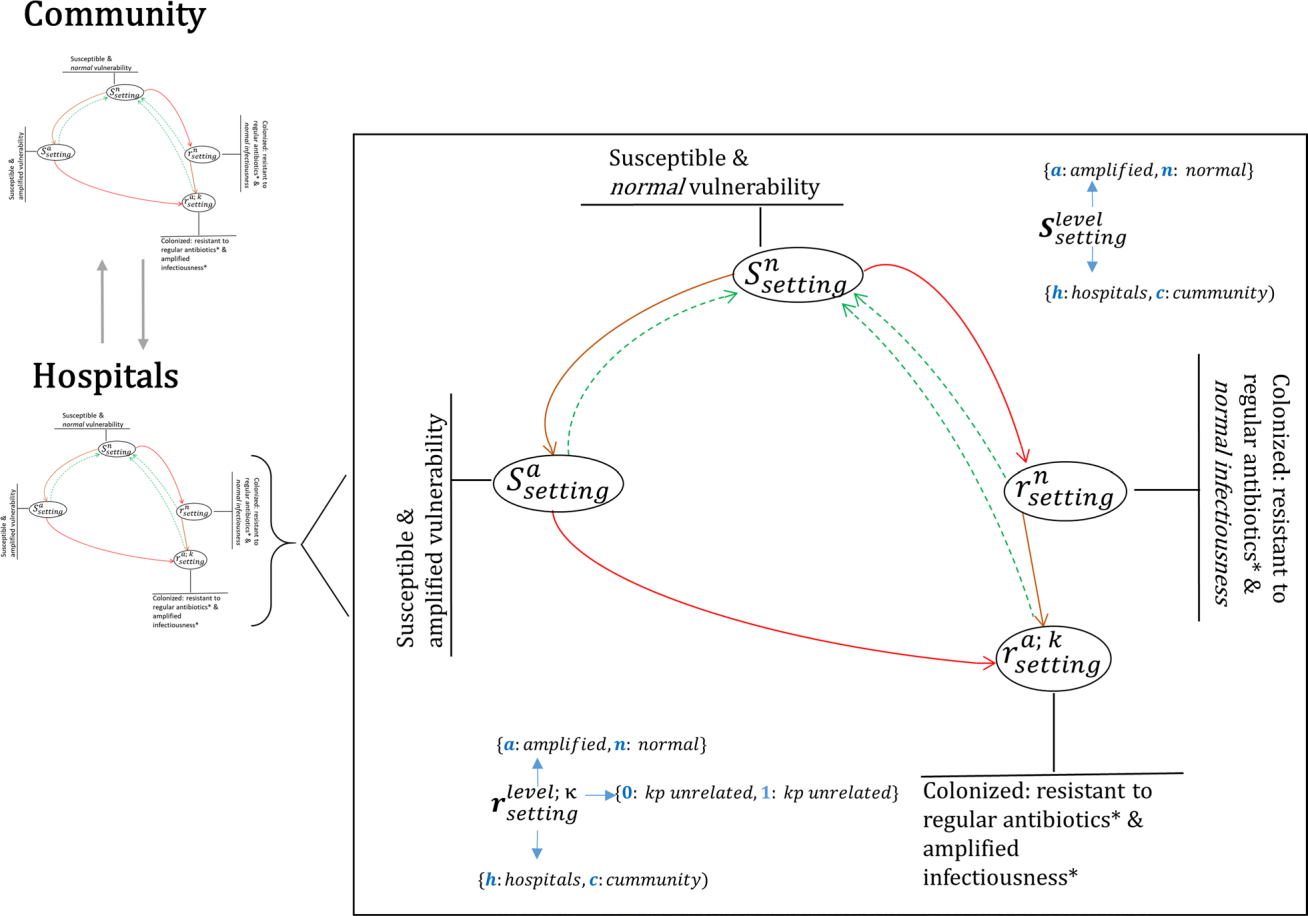
Simplified model structure. The model considers community and hospital settings interconnected by hospitalizations and dismissals. Transmission within each setting is simulated by means of a core model with setting specific parameters. In the core model (right black box), uncolonized individuals can have «normal» ($${S}^{n}$$) and «amplified» ($${S}^{a})$$ susceptibility to colonization, reflecting increased risk of colonization associated with antimicrobial therapy (dark, orange arrows). Colonization with ESBL-producing *Klebsiella pneumoniae* (red arrows) can occur through human-to-human contact locally (within hospitals and the community) and through external sources (e.g., traveling to high-prevalence areas and contaminated food). Colonized individuals are classified into two levels according to their ability to transmit the resistant pathogen: «normal» infectiousness ($${r}_{setting}^{n}$$) and «amplified» infectiousness $${r}_{setting}^{a,\kappa }$$ (dark, orange arrows). The model explicitly accounts for infections caused by ESBL producing *K. pneumoniae * with inadequate antimicrobial treatment $$(k=1$$, otherwise $$k=0$$). *ESBL-producing *K. pneumonia*

We developed a system of deterministic ordinary differential equations simulating the spread of colonization with ESBL-producing *K. pneumoniae* under the pressure of antimicrobial consumption in two settings, represented by two interconnected models: hospitals and community (Additional file 2). Figure 1 summarizes model structure. Model parameters were set to reflect interactions between settings, and their specific transmission dynamics. Table 1 shows the model parameters. The model assumes that a dynamic fraction of the population is hospitalized and that overall rates of antimicrobial consumption indicate a response to bacterial infections [18].

**Table 1:** Mathematical model parameters

Symbol	Description	Value	Source
Biological			
${\nu }_{S}$	Coefficient for amplified susceptibility to colonization after therapy with regular antimicrobial	3	[39]
${\nu }_{i}$	Coefficient for amplified infectiousness	2, sensitivity: 1, 3	[40]
ω	Susceptibility/infectiousness amplification factor for neutral antimicrobials with respect to regular ones	0.5	Assumption based on [40–42]
λ	Probability of clearing resistance to regular antimicrobials following treatment with restricted antimicrobials	0.8	Assumption (expert guess)
α	Rate of spontaneous clearance of colonization (year^−1^)^1^	1.4	[43, 44]
δ	Delay between end of treatment with restricted antimicrobials and complete resolution of colonization (months)	1	Assumption (expert guess)
1/${\varphi }_{c}$	Average time to spontaneous clearance of infection in the community (days)^2^	30	[45]
Transmission			
${\beta }_{h}$	human to human transmission colonization rate in hospitals (year^−1^)	10.9 (95% CI: 5.8–20.9)	Model fit
${\beta }_{c}$	human to human transmission colonization rate in the community (year^−1^)	0.67 (95% CI: 0.56–0.79)	Model fit
ε^3^	External force of colonization equivalent (% by 2015)	49.0^4^ (95% CI:6–100), sensitivity: 0–60%	Model fit; exogenous values
Antimicrobial consumption			
Treatment rate (treatments per year per inhabitant/patient, range)			
Hospital setting			
${\rm T}_{h,1}$	Regular antimicrobials	0.18–0.27	ANRESIS
${\rm T}_{h,2}$	Restricted antimicrobials	0.02–0.04	ANRESIS
${\rm T}_{h,3}$	Neutral antimicrobials	0.26–0.34	ANRESIS
		Community setting	
${\rm T}_{c,1}$	Regular antimicrobials	0.71 -0.80	ANRESIS
${\rm T}_{c,2}$	Restricted antimicrobials	0.0031–0.0032	ANRESIS
${\rm T}_{c,3}$	Neutral antimicrobials	1.5–1.71	ANRESIS
Average treatment duration (days)			
${\tau }_{1}$	Regular antimicrobials	8	Assumption based on clinical routine
${\tau }_{2}$	Restricted antimicrobials	5	Assumption based on clinical routine
${\tau }_{3}$	Neutral antimicrobials	8	Assumption based on clinical routine
* θ*	Hospitalisation rate	0.061–0.069	ANRESIS
μ_h_	Average length of hospitalization (days)	10	ANRESIS (set to reproduce data)
${\kappa }_{h/c}^{2}$	Fraction of infections resulting in antibiotic therapy that were caused by *Klebsiella pneumoniae* in hospitals/community^5^	7%	[46, 47]
${\rho }_{h}$	Fraction of infections caused by Klebsiella pneumoniae that were treated with restricted antibiotics (in hospitals)^6^	0.026	ANRESIS
${\rho }_{c}$	Fraction of infections caused by Klebsiella pneumoniae that were treated with restricted antibiotics (in the community)^6^	0.019	ANRESIS

^1^Average of values reported in the references

^2^Duration of colonization as proxy

^3^External force of colonization equivalent: Fraction of observed prevalence of colonization with ESBL-producing *Klebsiella pneumonia* atrributed to external sources

^4^Corresponds to a slope of increase in the external force of colonization of 0.13 [95% CI: 0.02 − 0.26] × 10 ^−3^ per year

^5^Average of fractions reported in the references

^6^Approximated by assuming that all invasive infections result in treatment with restricted antimicrobials after failure with regular ones

*Local transmission* Simulated persons hold a colonization status with ESBL-producing *K. pneumoniae* (colonized vs. non-colonized; compartments labelled *S* and *r*, respectively in Fig. 1). In the model, the result of local transmission events (e.g., “human-to-human”) is colonization with ESBL-producing *K. pneumoniae*. Transmission can occur in hospitals and in the community. The model considers setting-specific transmission rates.

*External force of colonization* This parameter aims to capture colonization through food products [19–21] and travellers returning from high-prevalence regions [22–24]. The model represents these processes through a “force of colonization”, independent of the prevalence within the modelled population. To reflect increasing prevalence worldwide and increasing number of people travelling to high-prevalence regions, we assumed a time dependent external force of colonization $\varepsilon \left(t\right)$, which increased at a constant speed from year 2000 onwards. To facilitate interpretation of this parameter, we refer to a figure derived from this rate, which we termed “external force of colonization equivalent” ε*(t). It approximates the proportion of prevalence observed until 2017 attributable to external sources ($\varepsilon * = \frac{1-{e}^{-{\int }_{2000}^{2017}\varepsilon \left(t\right)dt}}{P}, P$: $\mathrm{prevalence\, measured \,in }\,2017\,\mathrm{ in\, the \,community \,setting}$), when assuming exponentially distributed time to colonization).

*Effect of antimicrobial therapy on transmissions* We assumed that antimicrobial therapy increases the probability of becoming colonised, and a hypothetical analogous effect on the probability on onward transmission. The model therefore considered two levels of susceptibility and infectiousness: *normal* and *amplified* (Fig. 1, Table 1). Persons with *amplified* susceptibility (${S}^{a}$ in Fig. 1) were more likely to become colonized with ESBL-producing *K. pneumoniae* than those with *normal* susceptibility (${S}^{n}$) and were by analogy assumed to return to the state of normal susceptibility at a rate equal to that of spontaneous clearance. An a*mplified* susceptibility status was attained through antimicrobial treatment. Persons with *amplified* infectiousness (compartments ${r}_{setting}^{a,\kappa }$, where κ denotes resistance resulting from infections associated with *ESBL-producing K. pneumoniae* with inadequate antimicrobial treatment, see Fig. 1 and Additional file 2) were more likely to transmit ESBL-producing *K. pneumoniae*. *Amplified* infectiousness result from inadequate antimicrobial treatment. In the model, when a patient colonized with ceftriaxone-resistant *K. pneumoniae* was erroneously treated with regular antimicrobials for an infection caused by this pathogen, such treatment failed and was followed by treatment with restricted antibiotics. Successful treatment of such infections in hospitals blocked the possibility of onward transmission upon the patients’ return to the community setting.

### Model calibration and the role of external force of colonization

We estimated transmission rates for the hospital and community settings by fitting the model to reproduce the data on *K. pneumoniae* ceftriaxone susceptibility in both settings simultaneously. We also iteratively fitted and simulated transmission by assuming external rates of colonization equivalent ε* varying between 0 and 60% in steps of 15%. In an independent analysis, we estimated the external force of colonization resulting in the best model fit. We fitted the model by minimizing the sums of squared differences between model outputs and data points weighted to reflect measurement errors in the data.

The model was normalized to a constant population of 100,000 inhabitants, and the hospital setting represented as one single entity whose parameters reflected aggregated values across individual hospitals.

### Model projections on the impact of public health interventions

We considered interventions that would derive in different scenarios of antimicrobial consumption and in hospital transmission. Changes in these variables were modelled as exponential increases and declines at rates set to reach target levels in 2025.

*Modelled scenarios of antimicrobial consumption* Model projections considered hypothetical scenarios of change in: (1) overall consumption of antimicrobials, and (2) consumption of a restricted group of antimicrobials (carbapenems). Antimicrobial consumption was assumed to remain stable at current levels or to reach increases and decreases of 10%, 25% and 50% by 2025.

*Scenarios of in-hospital transmission* Hypothetical scenarios considered stable rates of in-hospital transmission (current levels) or increases and decreases of 10%, 25% and 50% by 2025.

### Sensitivity analyses on model projections:

*Infectiousness amplification* Because there are no reliable estimates for infectiousness amplification caused by antimicrobial consumption, we assessed the effect of a range of values for this parameter.

*External force of colonization* We assessed the robustness of our findings when confronted with extreme scenarios of contribution from external sources, represented in 60% and 0% equivalent external force of colonization.

All algorithms, including data processing, statistical analyses, solutions of differential equations, optimizations utilized in model fitting procedures, sensitivity and uncertainty analyses were implemented in R version 3.4.2 [25]. In particular, the packages *deSolve* [26], *optim* [27] and *FME* [28] were used for this study. We made all codes available on a public repository (https://github.com/svizcaya/amr-kleb).

## Results

Model calibration included data from susceptibility tests performed in 15,137 inpatients and 16,050 outpatients. Observed prevalence of ESBL-producing *K. pneumoniae* varied from 1.4% (95% CI: 0.4–3.7%) in 2005 to 10.4% (5.0–19.2%) in 2017 in the hospitals setting, and from 2.7% (0.7–7%) in 2007 to 2.4% (1.0–4.9%) in 2016 in the community setting. Consumption of all types of antimicrobials have increased in hospitals, reaching 55,747, 7,694 and 66,177 defined daily doses (DDD) for the *regular, restricted* and *neutral* types in 2015, respectively. Conversely, in the community, the use of *regular* and *neutral* antimicrobials changed only slightly over time, reaching 245,628 and 521,243 DDD in 2016 respectively, while consumptions of antimicrobials of the restricted type increased by 50% with respect to 2013, reaching 1150 DDD in the year 2015. Additional file 1: Fig. S1 shows these trends and, Additional file 1: Fig. S2 the corresponding rates and sizes of each contributing hospital.

**Fig. 2:**
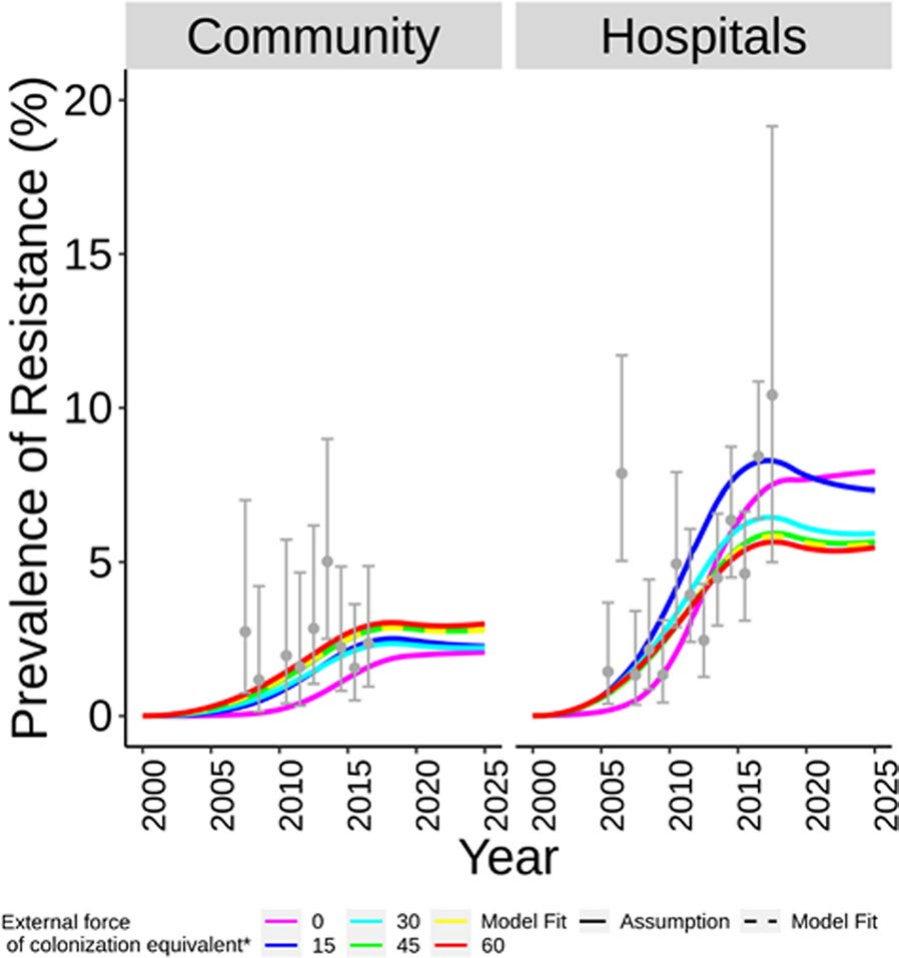
Measured prevalence, model fit and projections of colonization with *ESBL-producing Klebsiella pneumoniae* for a range of external forces of colonization. Data from ANRESIS (grey dots and error bars with 95% confidence intervals)**.** Projected future incidence in hospitals decreased monotonously with increasing external force of colonization. *The external force of colonization equivalent is a proxy for the fraction of observed prevalence by 2017 attributable to external sources

*Model calibration* Fig. 2 shows observed and modelled prevalence of ESBL-producing *K. pneumoniae*, and Table 1 the values for the fitted parameters. Data on prevalence in the community setting was only available from 2007 and unlike its hospital setting counterpart it did not suggest a clear trend. The model however assumed 2000 as the onset of transmission in both settings. This is in accordance with evidence of low prevalence in preceding years [13]. Estimated transmission rate in the hospital setting was 16-fold larger than that in the community setting. Fitted external force of colonization equivalent was 49.0 (95% CI: 6–100%). Modelled prevalence between 2005 and mid-2017 varied from 1.3% (95% CI: 0.6–2.1%) to 6.2% (5.1–7.6%) and from 0.6% (0.3–1.0%) to 3.2% (2.3–4.1%) in the hospital and community settings, respectively.

*Effect of the external force of colonization on prevalence* Fig. 2 also shows the effect of this parameter on model projections. Model fits that assumed increasing values for the external force of colonization resulted in lower, stabilizing future prevalence in hospitals. Maximum projected prevalence in hospitals by 2025, obtained by assuming null external force of colonization equivalent, was 7.8% (95% CI: 4.0–12.6%). Maximum projected prevalence in the community was 1.9% (0.7–4.4%), with little variation between scenarios of no null external force of colonization. These simulations assumed that antimicrobial consumption and transmission rates remained constant since 2018.

### Model projections on the impact of public health interventions between 2019 and 2025

**Fig. 3:**
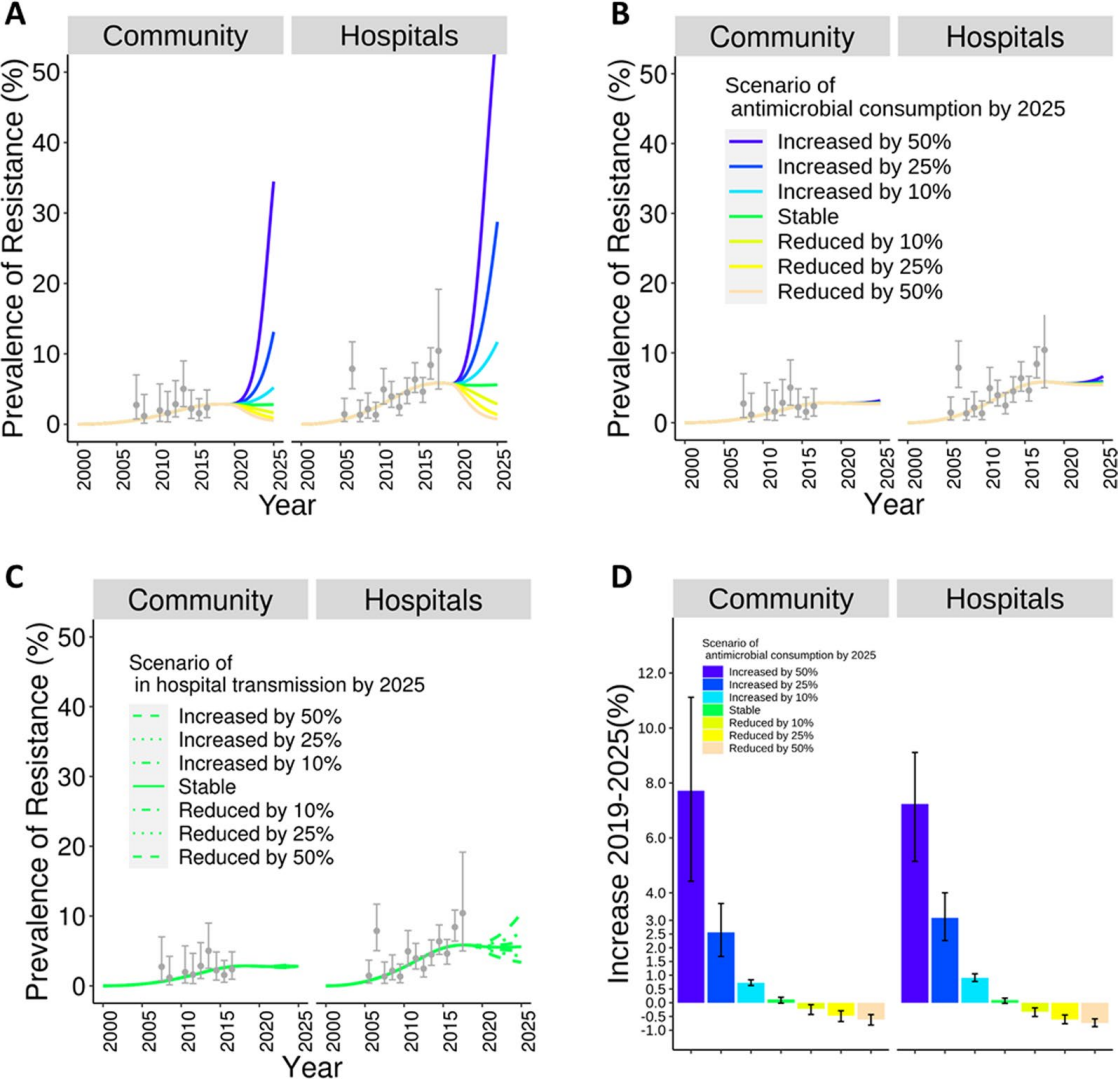
Projections of colonization with *ESBL-producing Klebsiella pneumoniae* for representative scenarios/strategies. Scenarios included changing: antimicrobial consumption (**A** ,**B** and **D**), and in-hospital transmission rate (**C**). In **A** scenarios of antimicrobial consumption included changes in all types of antimicrobials, while in **B** they included only antibiotics of the *carbapenem* class. **D** displays the comparison between the prevalence in 2019 and 2025 in **A**. The error bars show 95% confidence intervals over 243 iterations

Simulations assuming changes in overall antimicrobial consumption resulted in the largest changes in projected prevalence. It changed less in simulations considering changes only in carbapenem consumption than in simulations assuming changes in in-hospital transmission rates (Fig. 3).

### Scenarios of antimicrobial consumption

*Changes in overall antimicrobial consumption (Fig. **3**A)* Stable antimicrobial consumption led to almost unchanged prevalence between 2019 and 2025 in both settings. By contrast, future prevalence varied considerably across other scenarios of antimicrobial consumption, with more antimicrobials leading to rapid increases. A 50% increase in antimicrobial consumption led to a prevalence of 50.0% (95% CI: 46.5–58.1%) [eightfold increase from 6.1% (5.0–7.5%) in 2019] in hospitals and 26.4% (22.6–34.5%) in the community [eightfold increase from 3.2% (2.3–4.2%)] by 2025 (Fig. 3A). Analogously, although at a much lower speed, reductions in antimicrobial consumption led to declining prevalence. A 50% reduction in antimicrobial consumption resulted in a prevalence of 1.5% (0.7–3.1%; 75% reduction) in hospitals and of 1.2% (0.5–2.4%) (64% reduction) in the community by 2025. Figure 3D shows simulated changes in prevalence by 2025 with respect to 2019 across scenarios of overall antimicrobial consumption.

*Changes in carbapenem-class antimicrobial consumption (Fig. **3**B)* These simulations, which only varied consumption of carbapenem-class antimicrobials, resulted in changes in prevalence considerably smaller than those reported above did. When carbapenem consumption was set to increase by 50%, projected prevalence was 7.5% (95% CI: 6.4–9.8%) (17% increase) in hospitals and 3.9% (2.4–5.1%) (20% increase) in the community. When carbapenem consumption was set to decline by 50%, prevalence declined by less than 7% in both settings (Additional file 1: Fig. S3A).

*Scenarios of in-hospital transmission (Fig. **3**C)* Prevalence in 2025 almost doubled (6.1%; 95% CI: 5.0–7.5%) to 11.2% (8.9–14.1%) in the hospital setting and increased by 16% in the community when we assumed a 50% increase in in-hospital transmission rate. When in-hospital transmission rate was set to decline by 50%, prevalence dropped by 31% in hospitals and did not decline in the community (Additional file 1: Fig. S3B).

### Sensitivity analyses on the infectiousness amplification mediated by antimicrobials

The results of these analyses were similar to those of the main analysis. Additional file 1: Figs. S4-S7 are analogous to Figs. 2, 3 and Additional file 1: Fig. S3 when assuming no amplification (${\nu }_{i}$=1 versus ${\nu }_{i}$ =2 in the main analysis) and higher amplification (${\nu }_{i}$ = 3) of infectiousness after antimicrobial treatment with a *regular* agent. Prevalence projected in scenarios without amplification of infectiousness was higher and less sensitive to antimicrobial consumption than that projected in scenarios assuming higher amplification of infectiousness.

### Sensitivity analyses on the external force of colonization

The patterns were also robust to extreme scenarios of contribution from external sources of colonization (Additional file 1: Figs. S8-S9). Null external force of colonization led to projecting the lowest prevalence in the community and the highest in hospitals. The pattern inverts when assuming 60% equivalent external force of colonization. This further highlights the critical role the parameter in question to accurately project the future course of prevalence.

## Discussion

We developed a mathematical model that reconstructed the observed course of colonization with ESBL-producing *K. pneumoniae* between 2005 and 2017. The model assessed the potential comparative impact of changes in clinical practice on the future prevalence of ESBL-producing *K. pneumoniae*. The simulations suggested that with stable rates of antimicrobial consumption and in-hospital transmission, prevalence would stabilize in the near future. Former studies reached similar conclusions [29] and Swiss-wide data on antimicrobial resistance seems to be in line with this finding (Swiss Federal office of Public Health, Bulletin on antimicrobial # 52/2021). Estimated expected future prevalence depended on the assumed contribution of non-local transmission (*i.e.,* external forces of colonization). Increasing values for this parameter over the calibration period is bound to imply less local transmission, and led the model to project lower future prevalence in hospitals. This result highlights the importance of local interventions and the urgency for accurate data on the role of external sources. Simulated future scenarios showed that the most powerful driver of prevalence will be overall antimicrobial consumption, followed by in-hospital transmission. The influence of carbapenem consumption on prevalence stood evidently behind these two. Sustained stabilisation of antimicrobial consumption is however likely to require mediation of public health policies.

In line with published data [30], our model fits estimated transmission rates much higher within hospitals than in the community (16-fold). The best fit also suggested that as much as 49% of the prevalence of colonization measured until 2017 could be attributable to sources external to the modelled population, but uncertainty remained too wide to claim a finding. This uncertainty highlights the need for phylogenetic, timely evidence on the share of imported colonizations [31]. Data from a recent study from the Netherlands suggested a 43% prevalence of ESBL-producing *Escherichia coli* and *K. pneumoniae* among people who recently travelled to high-prevalence regions. In the same study, these travellers accounted for 18% of all cases of colonization with these pathogens [32]. Another study that screened retail raw vegetables in Amsterdam found ESBL-producing Enterobacteriaceae in 6% of screened samples [33].

These findings suggest that in low prevalence settings, an important share of colonizations with ESBL-producing Enterobacteriaceae could not be prevented by means of local public health interventions. Our model projections showed how three different interventions at the local level could influence future prevalence. For example, while an increase of only 10% in overall antimicrobial consumption may quickly trigger escalation of colonization, an equivalent reduction may lead to a sharp decrease in prevalence. The robustness of the results here outlined supports our modelling choices. Yet, a more precise description of the system could include effects of higher order influencing transmission. For instance, changes in the external force of colonization might be of a stochastic or cyclic nature.

Carbapenem prescriptions are currently intentionally restricted in routine clinical practice [34, 35]. In contrast, our model suggests that further reductions in consumption of the carbapenem-class alone would have a modest effect on the prevalence of ESBL-producing *K. pneumoniae*. Of note, mathematical models of pathogen transmission and at healthcare facility levels have investigated the role of antibiotic restriction and sequential treatment with different types of antimicrobials. Their results counterintuitively suggest that antibiotic restriction may promote resistance instead of hindering it [36, 37].

Our model was originally formulated to also model resistance to carbapenems. However, the setting we modelled did not have reported cases of carbapenem resistance over the study period. Our study did therefore not model the effects of increased carbapenem consumption on carbapenem resistance. This could limit the potential for generalization of our results to settings with prevalent carbapenemase resistance.

In line with former studies, the model was more sensitive to transmission rates than it was to single antibiotic class restrictions [38]. Simulations projected that eventual increases in in-hospital transmission rates could lead to considerable rises in prevalence in this setting. Reduced in-hospital transmission was effective at reducing prevalence within hospitals, but its effect on the community setting was relatively modest.

Although our findings remained qualitatively unchanged when confronted with different assumptions regarding increases in infectiousness of a resistant bacteria associated with antimicrobial therapy, the lack of estimates for this parameter in the literature may limit the scope of the values we projected for future prevalence. The sensitivity analyses showed that not only did this parameter, which reflects antimicrobial selection pressure, influenced the magnitude of the impact of antimicrobial consumption on prevalence; it was also decisive for the values of future prevalence even in the absence of changes in antimicrobial consumption. This indicates that precise estimates of future prevalence would require research aimed at estimating this parameter. Moreover, the concept of increased infectiousness is of hypothetical nature, further warranting such research.

We used the outcomes of susceptibility tests to ceftriaxone as a surrogate for presence/absence of ESBL-production. However, these tests are imperfect surrogate markers for the occurrence of ESBL resistances, as other mechanisms may also contribute to non-susceptibility.

The model was set to reproduce internally consistent time trends in outcomes of resistance tests, antimicrobial consumption and hospitalizations. Our findings regarding the comparative ability of different public health interventions to fight the spread of ESBL-producing *K. pneumoniae* are therefore likely to hold true for other regions.

### Implications of findings

These results can help inform public policy on strategies to mitigate the spread of resistant bacteria. Expectations regarding the impact of local interventions may need adjustment to account for constrains derived from potentially high contributions of non-local transmissions.

This study suggests that interventions including local or national antimicrobial stewardship programs might be most effective if they aim at reducing overall antimicrobial consumption. Further restricting antimicrobials of the carbapenem class is unlikely to noticeably decrease future prevalence of ESBL-producing *K. pneumoniae*. Therefore, in regions with low prevalence of carbapenem-resistant pathogens, additional restrictions of carbapenem must be carefully weighed against potential detrimental effects of an initially inappropriate antimicrobial therapy.

## Conclusion

Our simulations suggest that public health interventions reducing overall antimicrobial consumption would be considerably more powerful at reducing the prevalence of ESBL-producing *K. pneumoniae* than those reducing in-hospital transmissions, or further restricting carbapenem class antimicrobial consumption.

## Supplementary Information

**Additional file 1.** Supplementary file I.

**Additional file 2.** Supplementary file II.

## Data Availability

The datasets used and/or analysed during the current study are available from anresis.ch on reasonable request.
