# Supplementary material for: The impact of public health interventions on the future prevalence of ESBL-producing Klebsiella pneumoniae: a population based mathematical modelling study
Source: BMC Infect Dis. 2022 May 23;22:487. doi: 10.1186/s12879-022-07441-z (PMC9125893; doi:10.1186/s12879-022-07441-z)
Supplement: Supplementary file 1 — Additional file 1. Supplementary file I. [file 12879_2022_7441_MOESM1_ESM.docx]

Supplementary Material I

**The impact of public health interventions on the future prevalence**

**Of ESBL-producing *Klebsiella pneumoniae*:**

**a population based mathematical modelling study**

Luisa Salazar-Vizcaya *et. al.*,

**Table S1. Classification of antimicrobials according to categories in the model.**

| **Regular** | **Restricted** | **Neutral** |
| --- | --- | --- |
| Beta-lactamase resistant penicillins,  Beta-lactamase sensitive penicillins,  First-generation cephalosporins, Fluoroquinolones,  Fourth-generation cephalosporins,  Monobactams,  Penicillins with extended spectrum,  Second-generation cephalosporins and  Third-generation cephalosporins. | Carbapenems,  J01A and  Polymyxins | Antibiotics_Antimycotics for systemic use,  Antibiotics_Drugs for treatment of tuberculosis,  Combinations of penicillins, including beta-lactamase inhibitors,  Combinations of sulfonamides and trimethoprim, including derivatives,  Glycopeptide antibacterials, Hydrazides,  Imidazole derivatives_Antimycotics for systemic use,  Imidazole derivatives_Other antibacterials,  Trimethoprim and sulphonamide, not specified,  Lincosamides,  Macrolides,  Nitrofuran derivatives, not specified,  Other aminoglycosides, Other antibacterials,  Other antimycotics for systemic use,  Steroid antibacterials, Tetracyclines,  Triazole derivatives |

**SENSITIVITY ANALYSES ON THE INFECTIOUSNESS AMPLIFICATION MEDIATED BY ANTIMICRIBIALS**

**Model calibration (Figures S4**, analogous to Figure 2**):** Although the goodness of fit was very close to that in the main analysis, simulated future prevalence in hospitals was higher in most scenarios without amplification of infectiousness than it was in the main analysis (range in 2025: 6.4%-9.1% *versus* 5.2%-7.6% in hospitals and 2.2%-3.6% versus 2.0%-2.7% in the community respectively; **Figure S4A**). By contrast, in simulations assuming amplification of infection higher than that in the main analysis, future prevalence slowly declined and saturated near 2020.

**Figure S4. Measured prevalence, model fit and projections of colonization with *ESBL-producing Klebsiella pneumoniae* for varying external forces of colonization and assuming a higher coefficient of amplified infectiousness after incorrect antimicrobial therapy**

The coefficient of amplified infectiousness was **A)** **1** and **B) 3** (versus 2 in the main analyses). Projected future incidence in hospitals decreased monotonously with increasing external force of colonization.

Data from ANRESIS (grey dots and error bars with 95% confidence intervals).

*The external force of colonization equivalent is a proxy for the fraction of observed prevalence by 2017 attributable to external sources.


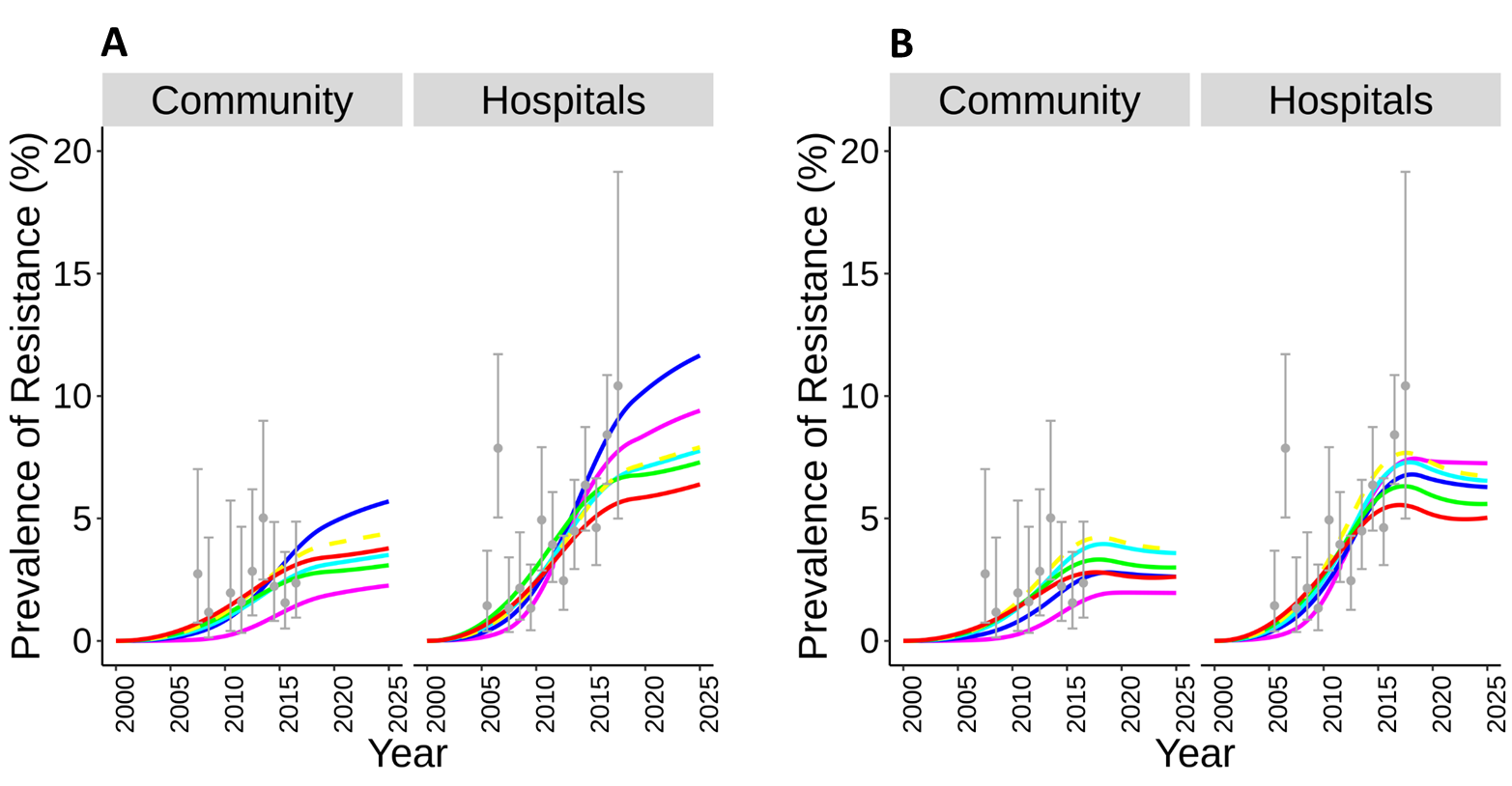


**Changes in antimicrobials consumption (Figures S5-S7**, analogous to Figures 3 and S3**):** As expected, the sensitivity of the model predictions to antimicrobial consumption increased with increasing infectiousness mediated by antimicrobials ($\nu_{i}$). **Changes in overall antimicrobials consumption (Figure S5A):** In simulations without increased infectiousness, a 50% increase in antimicrobials consumption was projected to lead to 6-fold increases in prevalence from 2019 in both hospitals and community settings (versus 8-fold increases in the main analysis; **Figures 3A** versus **S5A**). Analogously, a 50% reduction in antimicrobial consumption led to 60% and 49% reductions in hospitals and the community (versus 75% and 64% reductions in the main analysis; **Figure 3A** versus **Figure S5A**). **Changes in carbapenem-class antimicrobials consumption (Figures S5B , S6B, S7A and S7C):** In accordance with the main analysis, future prevalence in this analysis varied less than it did when overall antimicrobial consumption changed. When carbapenem consumption was set to increase by 50% in the scenario without amplification of infectiousness, projected prevalence in 2025 increased by 25% in both hospitals and the community settings (versus 20% and 17% increase in the main analysis), respectively (**Figures S5B and S7A**). Interestingly, when in the same scenario carbapenem consumption was set to decline by 50%, prevalence increased by 11% and 15% from 2019 (versus <7% reduction in the main analysis) in the hospitals and community setting, respectively. This suggests that the lower the ability of antimicrobials to amplify infectiousness, the less effective reductions in carbapenem consumption are at curbing prevalence.

**Scenarios of in-hospital transmission (Figures S5C, S6C, S7B and S7D):** As in the main analysis, simulated future prevalence was more sensitive to evaluated scenarios of transmission rate than to those considering changes in carbapenem consumption, and less sensitive than it was to overall antimicrobial consumption.

*Without amplified infectiousness* **(Figures S5C, S7B)**, prevalence in hospitals double between 2019 and 2025 when we assumed a 50% increase in in-hospital transmission rate. When in-hospital transmission rate was set to decline by 50%, prevalence in hospitals dropped by 31% (same as in the main analyses). As in the main analysis, prevalence in the community was insensitive to -hospital transmission. In this setting, prevalence increased by 36% and slightly increased by 8% (versus 16% and 3% increases in the main analysis) when in-hospital transmission was set to increase and decrease by 50%, respectively. With *high amplified* *infectiousness* **(Figures S6C, S7D)**, prevalence increased by 56% and 2% (versus 82% and 16% in the main analyses) in hospitals and community setting, respectively, when we assumed a 50% increase in in-hospital transmission rate. Prevalence likewise declined by 40% and 12% (versus 31% decline and a slight 3% increase of 0.1 percent points in the main analysis) in hospitals and the community, when in-hospital transmission rate declined by 50%. These analyses assumed that antimicrobial consumption remains stable in the future.

**ADDITIONAL SUPPLEMENTARY FIGURES**

**Figure S1. Observed antimicrobial consumption in hospitals and the community.**

**
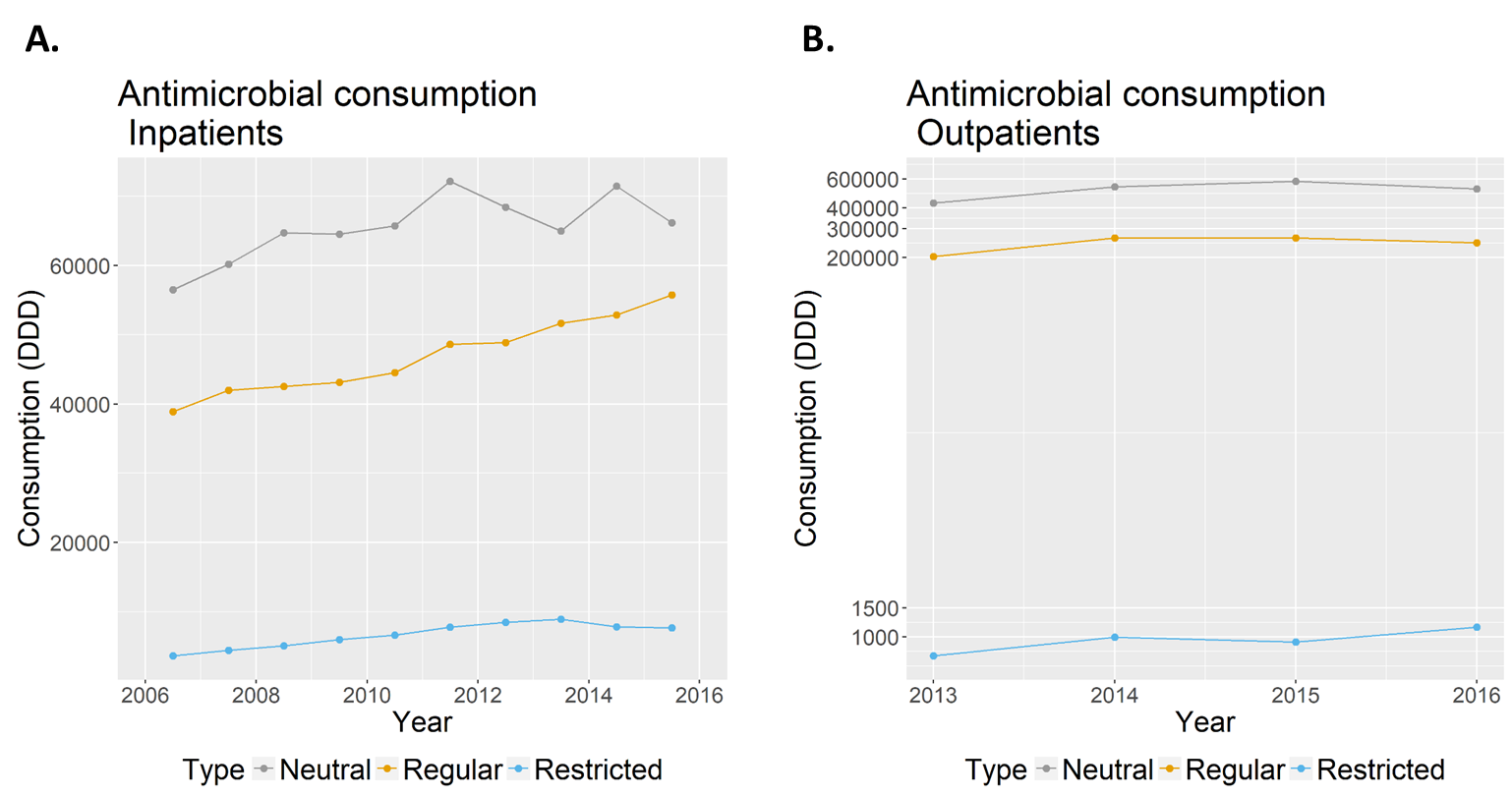
**

**Figure S2. Observed rates of antimicrobial consumption and sizes of the hospitals included for model calibration.**

**
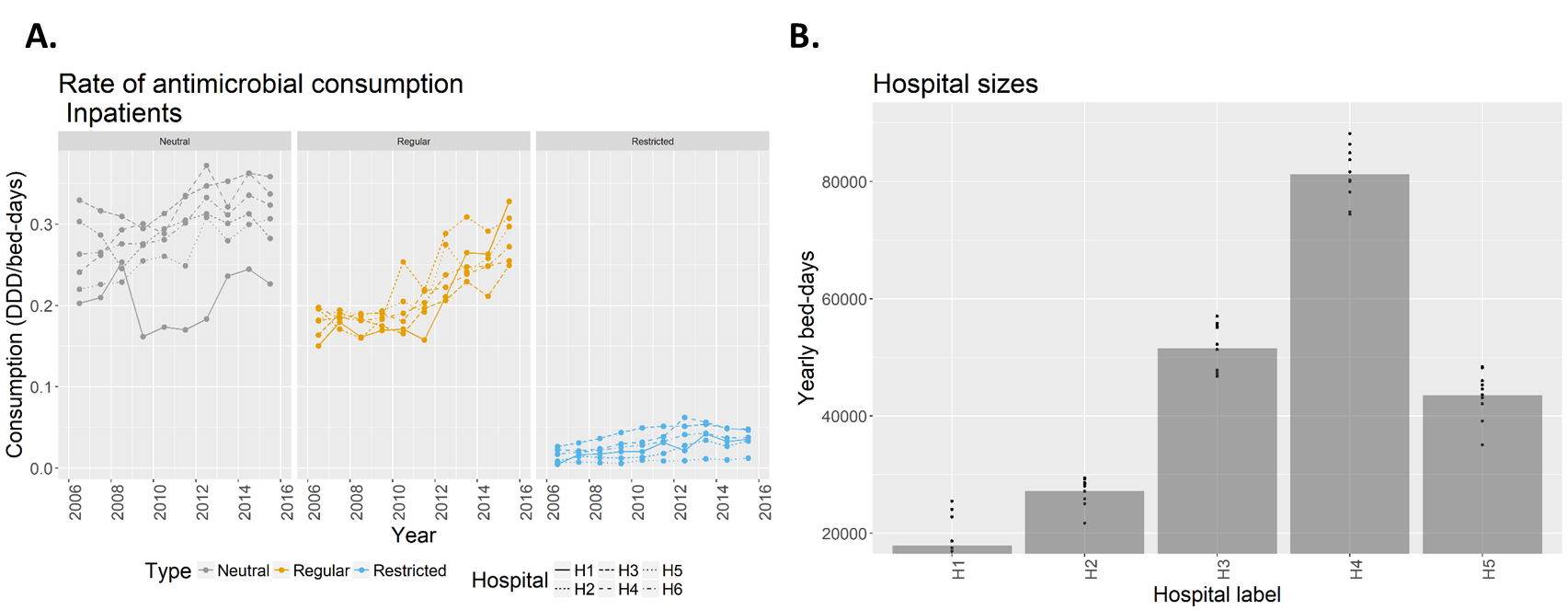
**

**Figure S3.** **Zooms-in of projections of colonization with *ESBL-producing Klebsiella pneumoniae* for representative scenarios/strategies**

**A)** Scenarios with changing: carbapenem consumption (zooms-in **Figure 3B**); and **B)** in-hospital transmission (zooms-in **Figure 3C**).


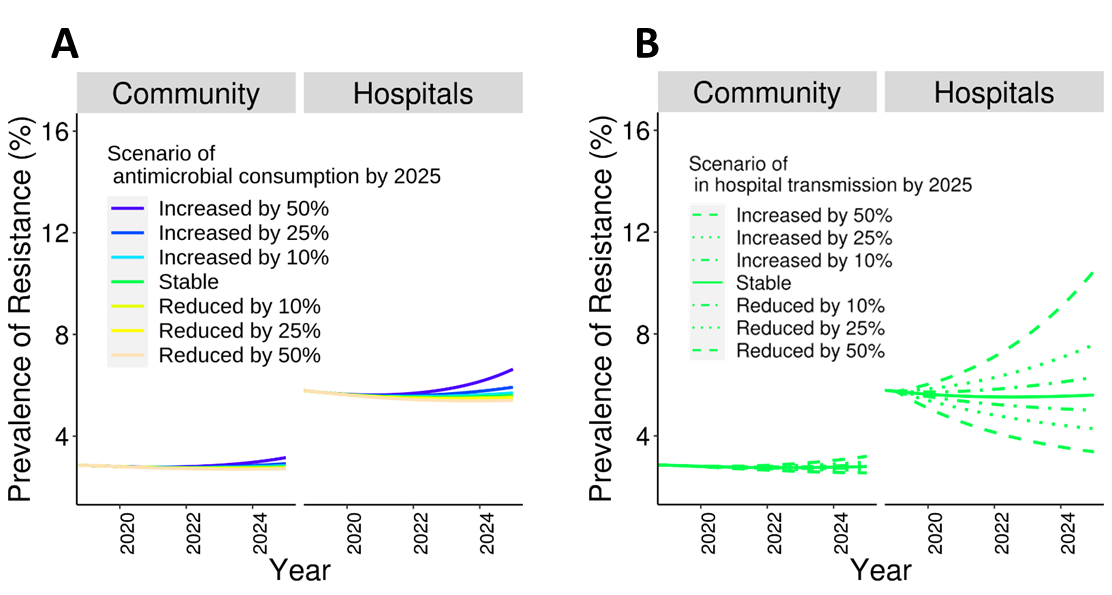


**Figure S5. Projections of colonization with *ESBL-producing Klebsiella pneumoniae* for representative scenarios/strategies assuming no amplified infectiousness after incorrect antimicrobial therapy**

The coefficient of amplified infectiousness was **1** (versus **2** in the main analyses). Scenarios included changing: antimicrobial consumption (**panels A** and **B**), and in-hospital transmission (**panel C**). In **Panel A** scenarios of antimicrobial consumption included all types of antimicrobials, while in **panel B** they included only carbapenem-class antimicrobials.


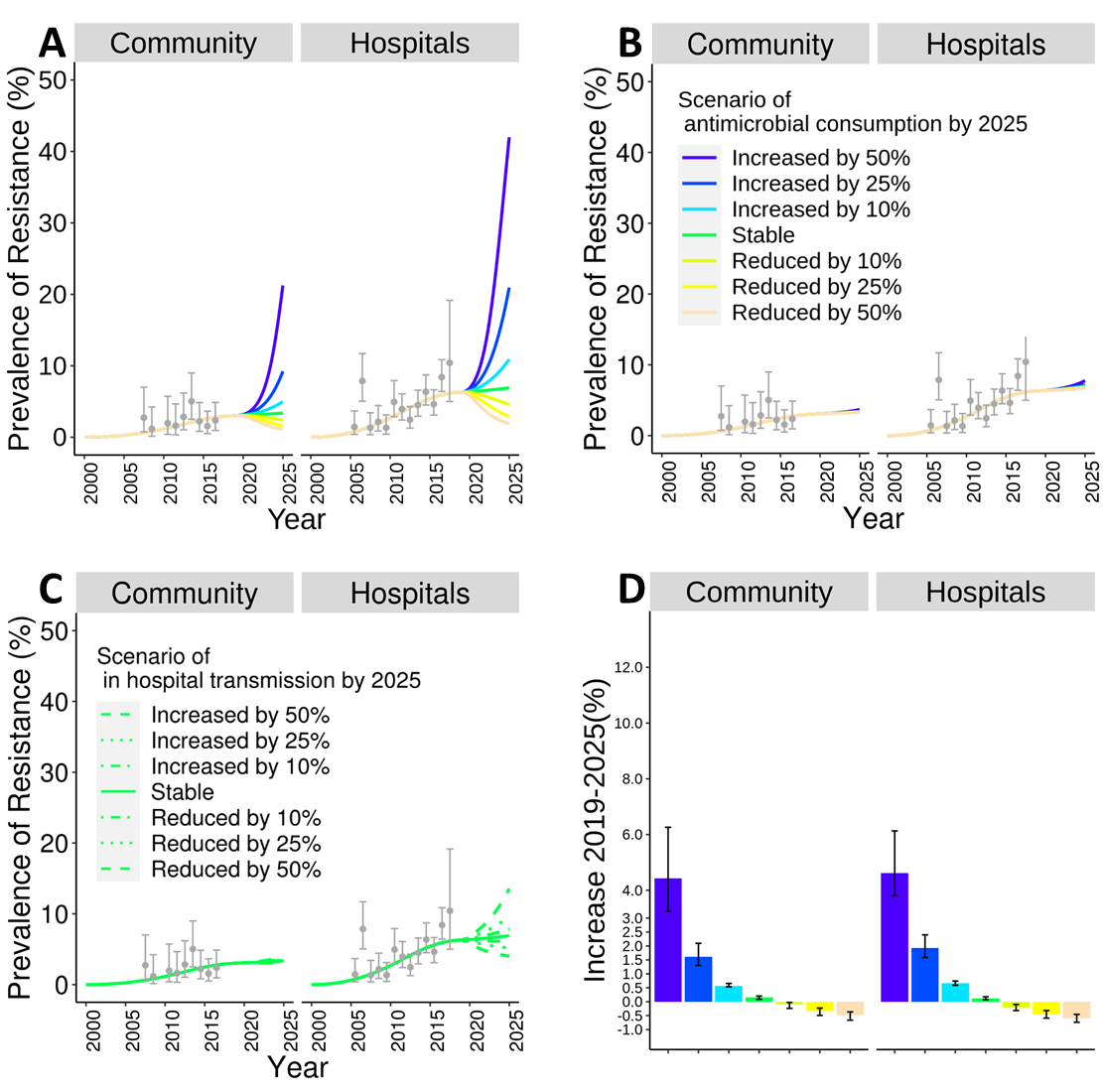


**Figure S6. Projections of colonization with *ESBL-producing Klebsiella pneumoniae* for representative scenarios/strategies assuming a lower coefficient of amplified infectiousness after incorrect antimicrobial therapy**

The coefficient of amplified infectiousness was **3** (versus **2** in the main analyses). Scenarios included changing: antimicrobial consumption (**panels A** and **B**), and in-hospital transmission (**panel C**). In **Panel A** scenarios of antimicrobial consumption included all types of antimicrobials, while in **panel B** they included only carbapenem-class antimicrobials*.*

*
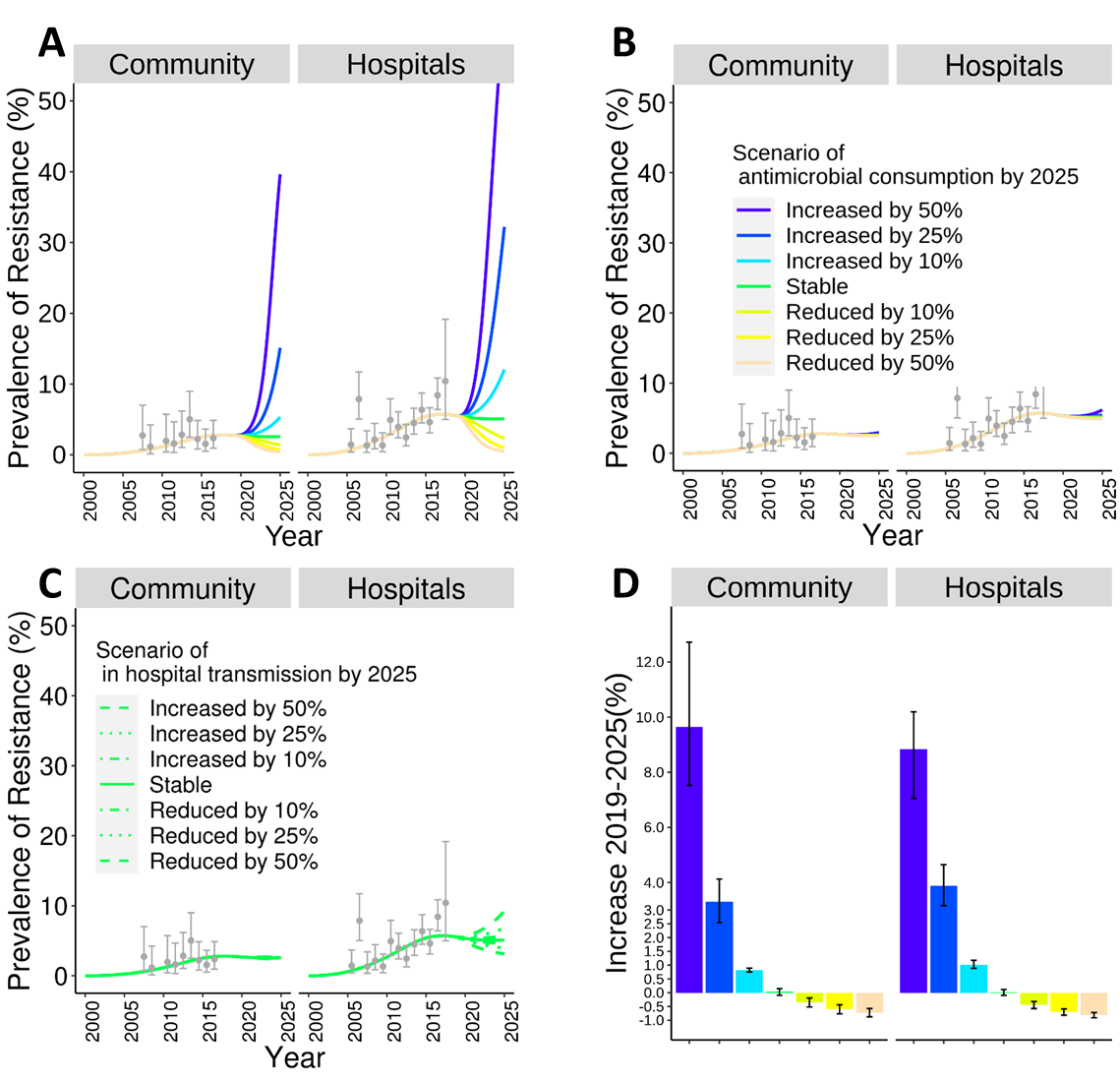
*

**Figure S7.** **Zooms-in of projections of colonization with *ESBL-producing Klebsiella pneumoniae* for representative scenarios/strategies and assuming a higher coefficient of amplified infectiousness after incorrect antimicrobial therapy**

The coefficient of amplified infectiousness was **A & B)** 1, and **C & D)** 3 (versus 2 in the main analyses). Scenarios with changing: carbapenem consumption (**panels A** and **C** zoom-in **Figures S5B and S6B**), and in-hospital transmission rate (**panels B** and **D** zoom-in **Figure S5C** and **S6C**).


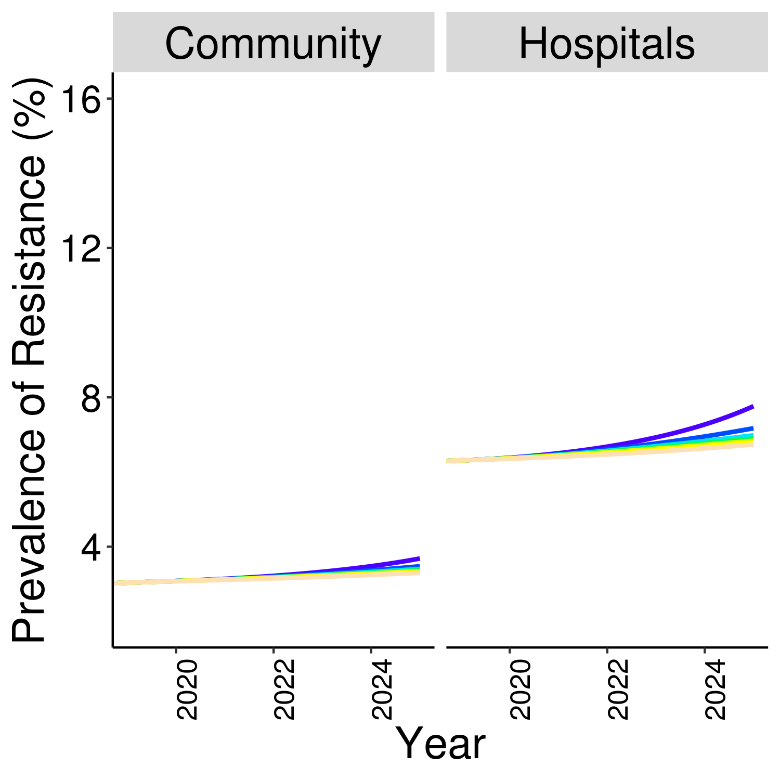

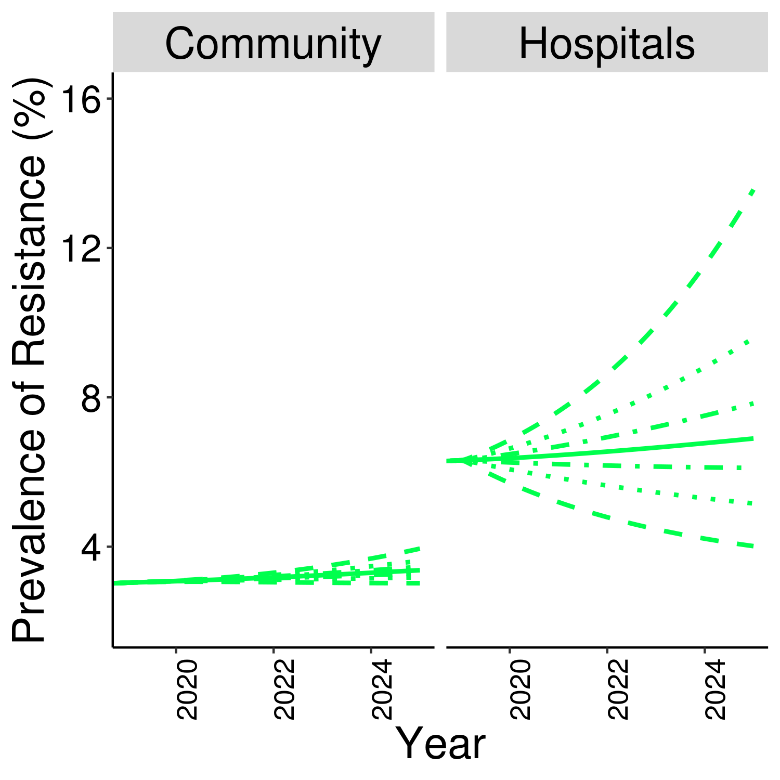


**A**

**B**


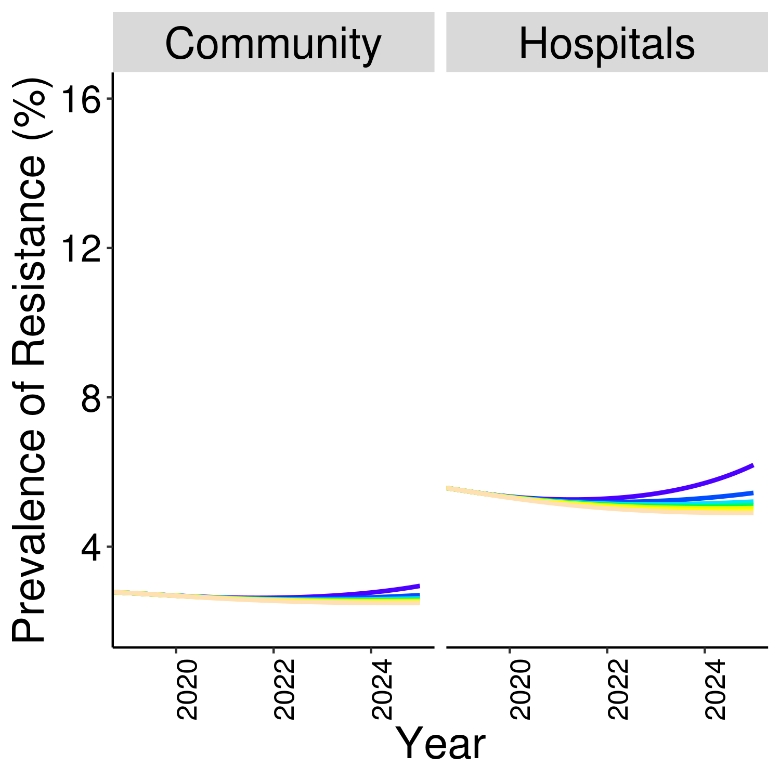

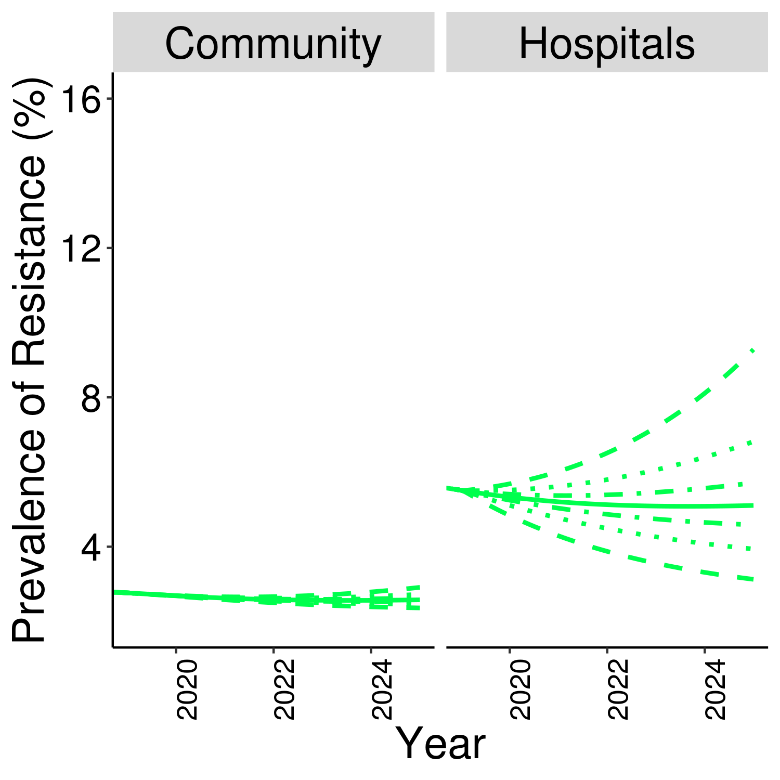


**C**

**D**


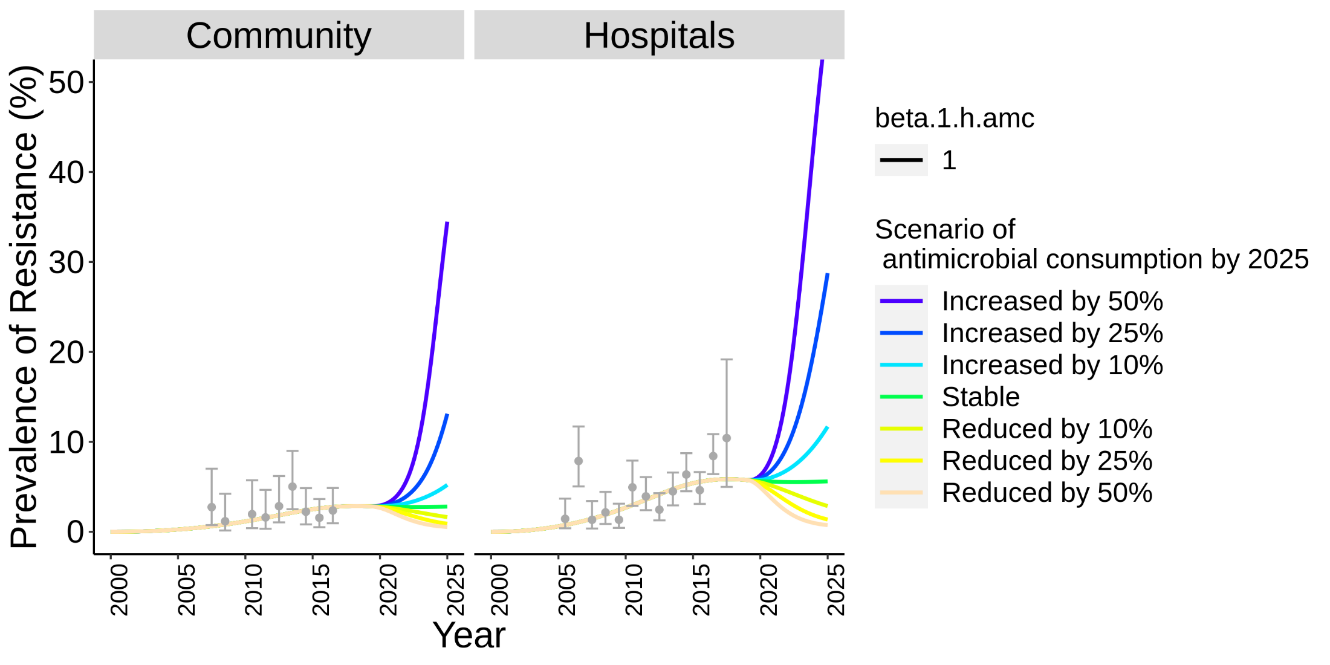

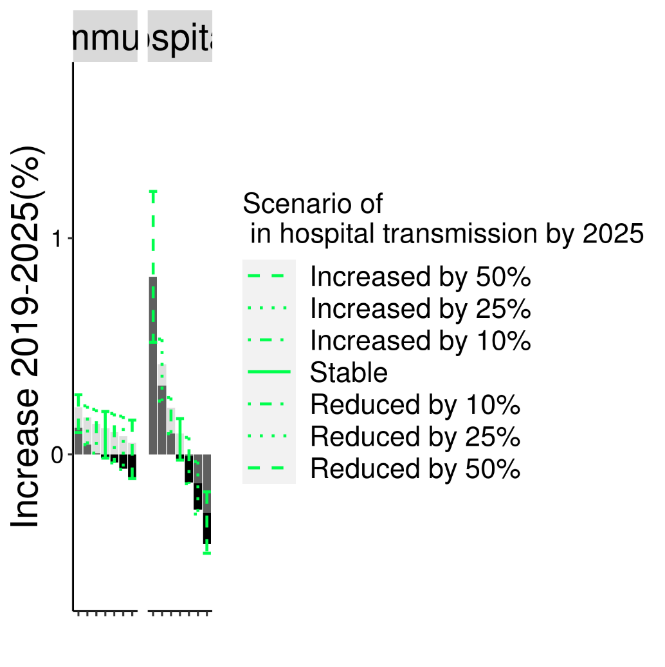


**Figure S8. Projections of colonization with *ESBL-producing Klebsiella pneumoniae* for representative scenarios/strategies for high (60%) external force of colonization equivalent.** ). Scenarios included changing: antimicrobial consumption (**panels A** and **B**), and in-hospital transmission (**panel C**). In **Panel A** scenarios of antimicrobial consumption included all types of antimicrobials, while in **panel B** they included only carbapenem-class antimicrobials*.*

*
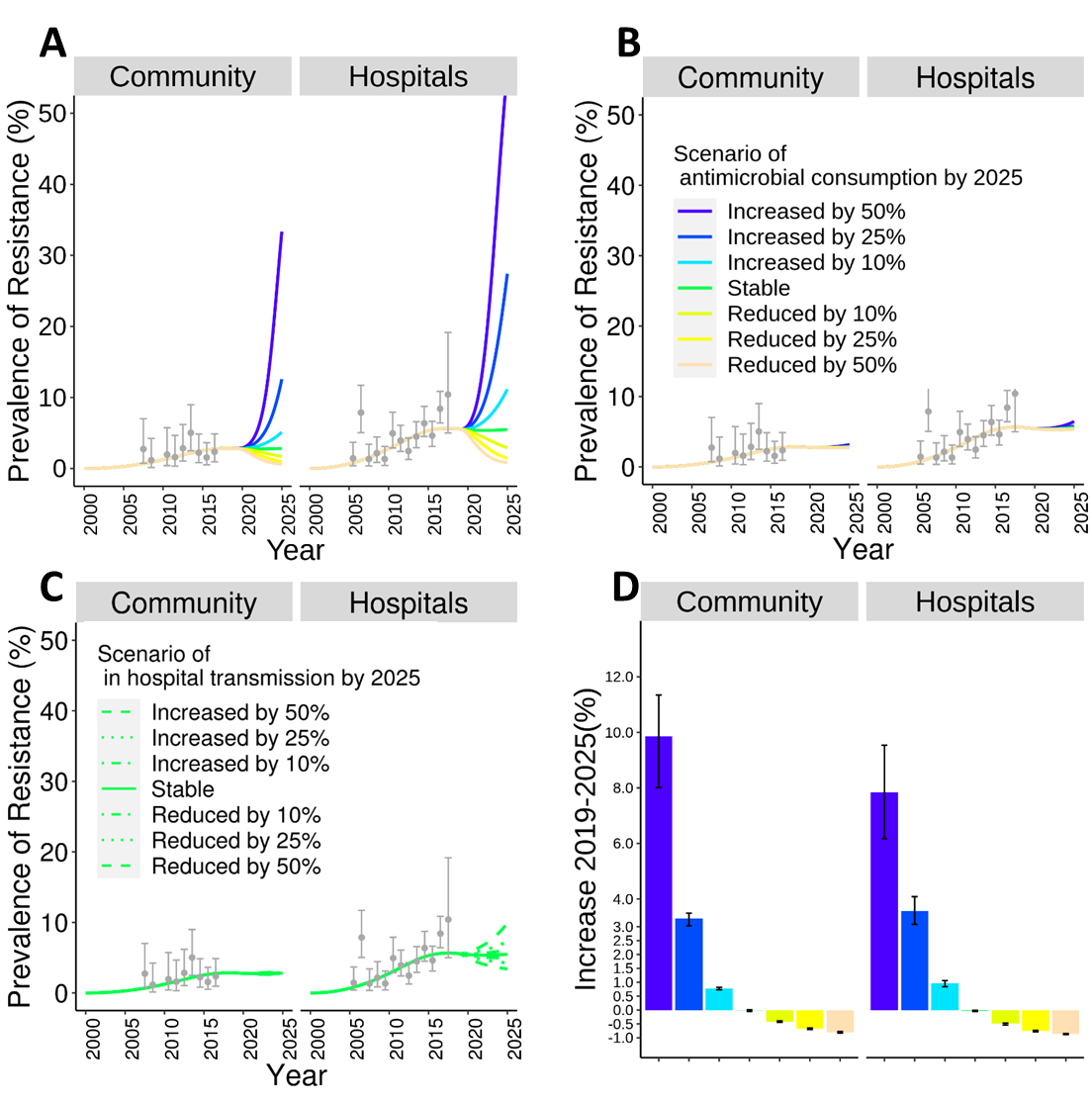
*

**Figure S9. Projections of colonization with *ESBL-producing Klebsiella pneumoniae* for representative scenarios/strategies without external force of colonization equivalent.** Scenarios included changing: antimicrobial consumption (**panels A** and **B**), and in-hospital transmission (**panel C**). In **Panel A** scenarios of antimicrobial consumption included all types of antimicrobials, while in **panel B** they included only carbapenem-class antimicrobials*.*

*
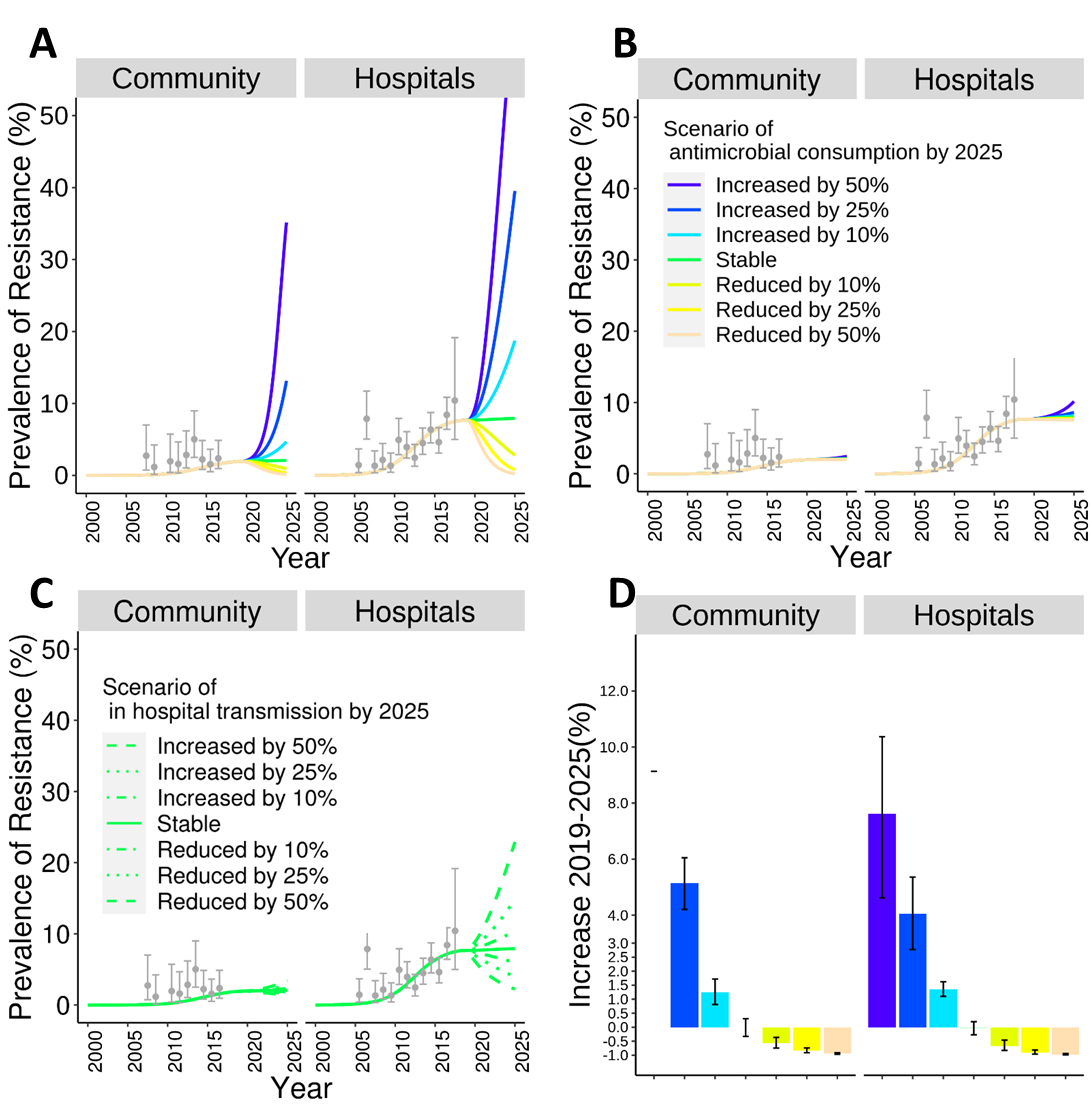
*
