# Supplementary material for: The impact of public health interventions on the future prevalence of ESBL-producing Klebsiella pneumoniae: a population based mathematical modelling study
Source: BMC Infect Dis. 2022 May 23;22:487. doi: 10.1186/s12879-022-07441-z (PMC9125893; doi:10.1186/s12879-022-07441-z)
Supplement: Supplementary file 2 — Additional file 2. Supplementary file II. [file 12879_2022_7441_MOESM2_ESM.pdf]

Supplementary Material II: Model equations

The impact of public health interventions on the future prevalence of *ESBL-producing Klebsiella pneumoniae:* a population based mathematical modelling study

Luisa Salazar-Vizcaya, et.al

$S_{setting}^{level}$: level $\rightarrow$ $\{a: amplified, n: normal\}$; setting $\rightarrow$ $\{h: hospitals, c: cummunity)$

$r_{setting}^{level;\,\kappa}$: level $\rightarrow$ $\{a: amplified, n: normal\}$; κ $\rightarrow$ $\{0: kp\ unrelated, 1: kp\ unrelated\}$; setting $\rightarrow$ $\{h: hospitals, c: cummunity)$

$T_{setting,\,type}$: type $\rightarrow$ $\{1: regular, 2: restricted\ 3: neutral\}$; setting $\rightarrow$ $\{h: hospitals, c: cummunity)$

Model equations

$$\dot{S}_{setting}^{n} = (-1)^{\eta}\left(\theta(t) S_{setting=community}^{n} - \mu_h S_{setting=hospitals}^{n}\right) - \frac{1}{N_{setting}}\beta_{setting}\left(r_{setting}^{n} + \nu_i \sum_{\kappa\in\{0,1\}} r_{setting}^{a;\kappa}\right) S_{setting}^{n} - \sum_{type\in\{1,2,3\}} T_{setting,type}(t)\omega^{[type/3]} S_{setting}^{n} + T_{setting,type=2}(t)\lambda\left(r_{setting}^{n} + r_{setting}^{a;\kappa=0}\right) + \alpha_{setting}\left(S_{setting}^{a} + r_{setting}^{n} + \sum_{\kappa\in\{0,1\}} r_{setting}^{a;\kappa}\right) - \epsilon(t) S_{setting}^{n} + \lambda\left(T' - \phi_{setting}\right) r_{setting}^{a;\kappa=1}$$

$$\dot{S}_{setting}^{a} = (-1)^{\eta}\left(\theta(t) S_{setting=community}^{a} - \mu_h S_{setting=hospitals}^{a}\right) - \frac{1}{N_{setting}}\beta_{setting}\left(r_{setting}^{n} + \nu_i \sum_{\kappa\in\{0,1\}} r_{setting}^{a;\kappa}\right) S_{setting}^{a}\nu_s + \sum_{type\in\{1,2,3\}} T_{setting,type}(t)\omega^{[type/3]} S_{setting}^{n} - \alpha_{setting} S_{setting}^{a} - \epsilon(t) S_{setting}^{a}\nu_s$$

$$\dot{r}_{setting}^{n} = (-1)^{\eta}\left(\theta(t) r_{setting}^{n} - \mu_h r_{setting=hospitals}^{n}\right) + \frac{1}{N_{setting}}\beta_{setting}\left(r_{setting}^{n} + \nu_i \sum_{\kappa\in\{0,1\}} r_{setting}^{a;\kappa}\right) S_{setting}^{n} - \sum_{type\in\{1,2,3\}} T_{setting,type}(t)\omega^{[type/3]} r_{setting}^{n} - T_{setting,type=2}(t)\lambda r_{setting}^{n} - \alpha_{setting} r_{setting}^{n} + \epsilon(t) S_{setting}^{n}$$

$$\dot{r}_{setting}^{a;\kappa=1} = (-1)^{\eta}\left(\theta_h(t) S_{setting}^{a} - \mu_h r_{setting=hospitals}^{a;\kappa=1}\right) + \sum_{type\in\{1,2,3\}} T_{setting,type}(t)\omega^{[type/3]} r_{setting}^{n}\kappa - \alpha_{setting} r_{setting}^{a;\kappa=1} - \lambda\left(T' - \phi_{setting}\right) r_{setting}^{a;\kappa=1} - \phi_{setting} r_{setting}^{a;\kappa=1}$$

$$\dot{r}_{setting}^{a;\kappa=0} = (-1)^{\eta}\left(\theta(t) S_{setting}^{a} - \mu_h r_{setting=hospitals}^{a;\kappa=0}\right) + \frac{1}{N_{setting}}\beta_{setting}\left(r_{setting}^{n} + \nu_i \sum_{\kappa\in\{0,1\}} r_{setting}^{a;\kappa}\right) S_{setting}^{a}\nu_s + \sum_{type\in\{1,2,3\}} T_{setting,type}(t)\omega^{[type/3]} r_{setting}^{n}(1-\kappa) - T_{setting,type=2}(t)\lambda r_{setting}^{a;\kappa=0} - \alpha_{setting} r_{setting}^{a;\kappa=0} + \epsilon(t) S_{setting}^{a}\nu_s + \phi_{setting} r_{setting}^{a;\kappa=1}$$

Where:

- Superscripts n : *normal* and a : *amplified* represent levels of susceptibility to and infectiousness of colonization.
- Subscript $setting = \{hospitals, community\}$
- $\kappa = 1$ indicates resistance associated with erroneous treatment of an infection associated with *ESBL-producing Klebsiella pneumoniae*
- $N_{setting}$ denotes the total population in each setting
- $\eta = \begin{cases} 0, & \text{if } setting = h \\ 1, & \text{if } setting = c \end{cases}$
- $\kappa = 1$ indicates resistance associated with erroneous treatment of an infection associated with *ESBL-producing Klebsiella pneumoniae*
